# The Glycosaminoglycan-Dependent Interactome of Neurexin-1 in Human Fetal Glial Cells

**DOI:** 10.1002/pgr2.70039

**Published:** 2025-10-12

**Authors:** Meg Critcher, Han Wu, Yajing Lu, Mia L. Huang

**Affiliations:** Department of Chemistry, Scripps Research, La Jolla, California, USA

**Keywords:** glycosaminoglycan, interactome, neurexin-1, proximity tagging, thrombospondin-1

## Abstract

Neurexins are synaptic adhesion molecules best characterized in neurons, where they regulate synapse assembly and function, with emerging evidence indicating they are also abundantly expressed by astrocytes. To elucidate the interactome of NRXN1α, we employed a proximity labeling strategy in cultured human fetal glial cells (SVG p12 cells). This approach enables the identification of transient and spatially restricted protein interactions, offering insights into the molecular environment of NRXN1α in glia. Further, we investigated how the presence and number of glycosaminoglycan (GAG) chains present on NRXN1α influence these interactions by generating glycosylation-deficient mutants at previously characterized GAG glycosites. Here, we show that the astrocytic NRXN1α interactome in SVG p12 cells consists of over 400 proteins, half of which are likely modulated by GAGs. Our findings provide a systems-level view of NRXN1α-associated proteins in fetal glia cultured in the absence of neurons and highlight the role of GAG valency in modulating its interactome.

## 1 | Introduction

The majority of excitatory synapses in the brain are considered tripartite, consisting of presynaptic and postsynaptic neuronal elements along with fine perisynaptic astrocytic processes [1, 2]. Astrocyte-synapse associations are thought to regulate ion homeostasis, neurotransmitter recycling, synapse development—including formation, maturation, and elimination—and synaptic plasticity [3–7]. Recent studies have implicated astrocytic cell adhesion molecules in regulating synapse and astrocyte development, including NrCAM, HepaCAM, and N-cadherin [8–10]. The observation that these neuronal proteins are also being expressed by supporting astroglial cells paints a dynamic image of cell surface proteins within the tripartite synapse.

The “neurexin code” refers to the abundance of splice and structural variants within the neurexin family. Further, it proposes that these species form a molecular code that specifies synapse assembly and function across diverse types of synapses [11, 12]. Indeed, various animal models have identified critical roles for neurexins in synapse assembly and neurotransmission [11–13]. Each of the three *NRXN* genes is expressed as α-neurexin and β-neurexin from independent promoters, with NRXN1 possessing an additional short γ isoform. Further diversity is introduced by 5–6 canonical splice sites (SS1–6, of which SS1–3 and SS6 are specific to α-neurexin [14, 15]), which can give rise to thousands of NRXN variants [16]. Of these splice sites, SS4 and to a lesser extent SS2, have been well-characterized and shown to modulate interactions with canonical NRXN ligands [11, 12, 17, 18]. It was also recently discovered that neurexins can be post-translationally modified with heparan sulfate (HS) in disordered regions within their extra-cellular domain [19], with the inclusion of SS5 producing an additional site of HS attachment [20]. This discovery adds further structural and functional diversity to the neurexin code due to the polydisperse and sulfated nature of HS as a glycosaminoglycan (GAG), which forms a wide range of interactions with various biomolecules [21–23]. Further, these discoveries define NRXNs as proteoglycans (PGs) [24].

PGs are a unique form of protein glycoconjugate, defined by the modification of a peptide backbone with linear, polysulfated GAG chains, typically in the form of HS or chondroitin sulfate (CS). Indeed, some PGs can possess both chains on a single peptide backbone to form hybrid PGs [25], such as syndecan-1 [26]. As it has been estimated that ~74% of syndecan interactions involve GAG chains [27], this modification likely presents an understudied aspect of the neurexin code. Understanding its interactome with consideration of its architecture as a PG (protein isoforms, GAG occupancy, composition, and sulfation [28]) is critical to decode neurexin functions. Further, astrocytic secretion of the glypican family of HS-modified PGs has been established as key for synapse formation and maturation [29–31]. Such studies have established a role for PGs in astrocytes, which we hypothesize will extend to GAG-modified NRXN1.

Neurexins and their ligands (e.g., neuroligins) have longstanding links to various neuropsychiatric disorders, and GAG valency on NRXN1 has been implicated in autism-associated behaviors [20]. As such, solving the interactome of NRXN1α may identify additional players in neurodevelopmental and psychiatric disorders [32]. Although NRXN studies have primarily concentrated on their roles as neuronal presynaptic proteins, a recent preprint revealed that NRXN1α is abundantly expressed by astrocytes, where it exhibits significantly greater GAG modification compared to neurons [33]. Here, we designed an APEX2 fusion construct [34] with NRXN1α (PX-NRXN1α) to perform proximity labeling in cultured human fetal astroglial cells. Further, we mutated previously characterized GAG sites to create PX-NRXN1α glycosylation mutants to elucidate the effects of glycosylation on the neurexin interactome in glial cells. We and others have demonstrated the ability of APEX2-fused constructs to capture glycan- and GAG-dependent interactions in live cells by proximity labeling [35–39]. Here, we utilized the fetal astroglial cell line SVG p12 [40, 41], which resembles glial progenitor cells, to define the glial NRXN1α interactome and the impact of GAG valency. Using this approach, we show that > 400 proteins are significantly enriched by PX-NRXN1α and labeling with a GAG-null mutant substantially decreases the enrichment of many proteins.

## 2 | Results

### 2.1 | Design and Characterization of PX-NRXN1α Expression Constructs

**FIGURE 1 |:**
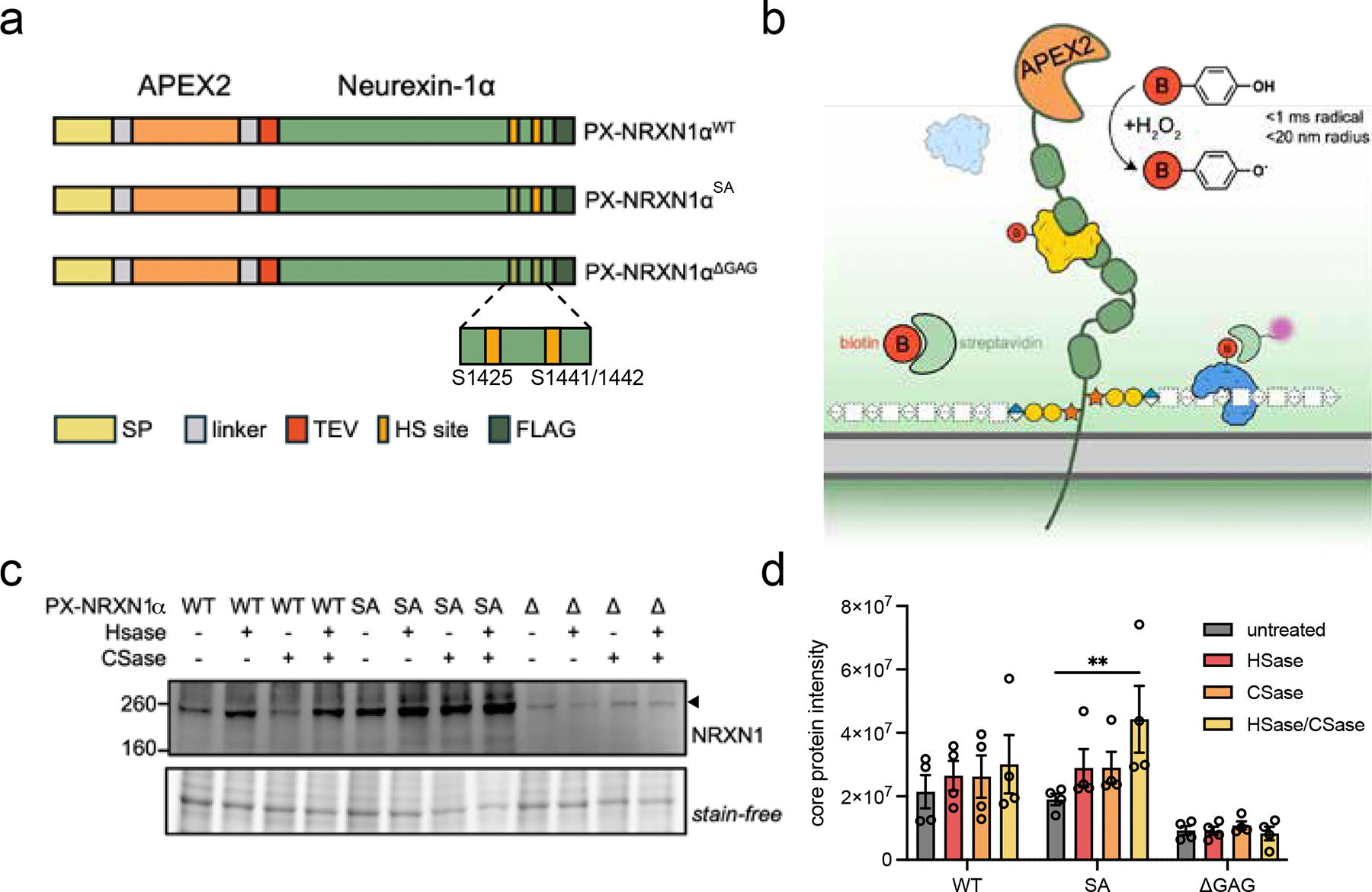
Overexpression of PX-NRXN1α and HS-deficient mutants in astrocyte-like cells. (a) Cartoon depiction of APEX2-NRXN1α proteins, including a signal peptide (SP) to promote membrane localization, flexible SSGS linker, Tobacco Etched Virus (TEV)-cleavable linker, two GAG attachment sites (S1425 and S1441/S1442) mutated to alanine, and C-terminal FLAG tag. (b) Cartoon depiction of proximity labeling in PX-NRXN1α-expressing cells. Cells are incubated with biotin phenol (red), which forms a biotin-phenoxyl radical upon addition of H_2_O_2_, catalyzed by APEX2 (orange), which tags nearby proximal proteins. (c) Western blot analysis of lysates collected from transfected cells reveals smear-like patterns in PX-NRXN1α^WT^ (“WT”) and PX-NRXN1α^SA^ (“SA”), which are absent in the ΔGAG (“Δ”) mutant. Incubation of lysates with heparinase I/II/III (HSase) and/or chondroitinase ABC (CSase) results in a clear enhancement of core protein band intensity (~260 kDa, black arrow. (d) Quantification of PX-NRXN1α protein core band intensity. Data are representative of four biological replicates. One-way analysis of variance (ANOVA). Bar graphs represent means, and error bars represent SEM.

To probe the interactome of NRXN1α, we designed mammalian expression plasmids with an N-terminal APEX2 peroxidase fused to human NRXN1 isoform α2 (PX-NRXN1α^WT^, Figure 1a). This isoform was selected as it contains the additional three amino acids that confer a secondary GAG site, allowing us to explore the role of valency in the NRXN1α interactome [20]. As such, we prepared glycosylation mutants as previously published—a single mutant S1425A [19] (PX-NRXN1α^SA^) and a triple mutant with the S1425A mutation and two mutations adjacent to SS5, S1441A/S1442A [20] (PX-NRXN1α^ΔGAG^, Figure 1a). To direct our constructs to the plasma membrane, the signal peptide of NRXN1α was moved to the N-terminus of our protein, ahead of APEX2 (Figure 1a). Prior studies have identified HS at both GAG sites in neurons; however, consistent with prior findings of increased NRXN1 HS valency in astrocytes [33], we postulate that NRXN1 glycosylation may be cell-type dependent and have a major impact on its interactome. Thus, to explore the interactome of NRXN1α in astrocytes, we expressed our APEX2 fusion proteins for proximity labeling in the fetal astroglial cell line SVG p12 (Figure 1b), which does not express detectable amounts of endogenous NRXN1 (Supporting Information S1: Figure S1a).

The expression of PX-NRXN1α^WT^ results in a high molecular weight band migrating at ~260 kDa as a smear, indicative of a polydisperse GAG-modified protein (Figure 1c, Supporting Information S1: Figure S1a,b). To determine which GAG species PX-NRXN1α is modified with in SVG p12 cells, cell lysates expressing PX-NRXN1α variants were incubated with a cocktail of bacterial heparinase I/II/III (HSase) or chondroitinase ABC (CSase) enzymes to cleave HS or CS, respectively. Western blot analysis reveals an enhancement in the band intensity for the core protein band upon treatment of PX-NRXN1α^WT^ with HSase alone or co-treatment with CSase, suggesting this protein is a hybrid (HS and CS modified) PG (Figure 1c,d). As expected, no substantial changes were observed with the triple mutant PX-NRXN1α^ΔGAG^, confirming the lack of GAG chains on this protein (Figure 1c,d). We also note that this protein is expressed at lower levels than other constructs (Supporting Information S1: Figure S1c). The observation that only PX-NRXN1α^SA^ appears to be substantially modified by both HS and CS suggests that in the absence of glycosylation at the canonical HS site [19] (S1425), the SS5 introduced GAG site may be a hybrid site that can be modified with either HS or CS chains. This hypothesis is supported by previous observations where HSase treatment did not fully reduce the presence of high molecular weight NRXN1α [19, 33].

### 2.2 | The Extent of PX-NRXN1α-Mediated Proximity Labeling Is GAG-Dependent

**FIGURE 2 |:**
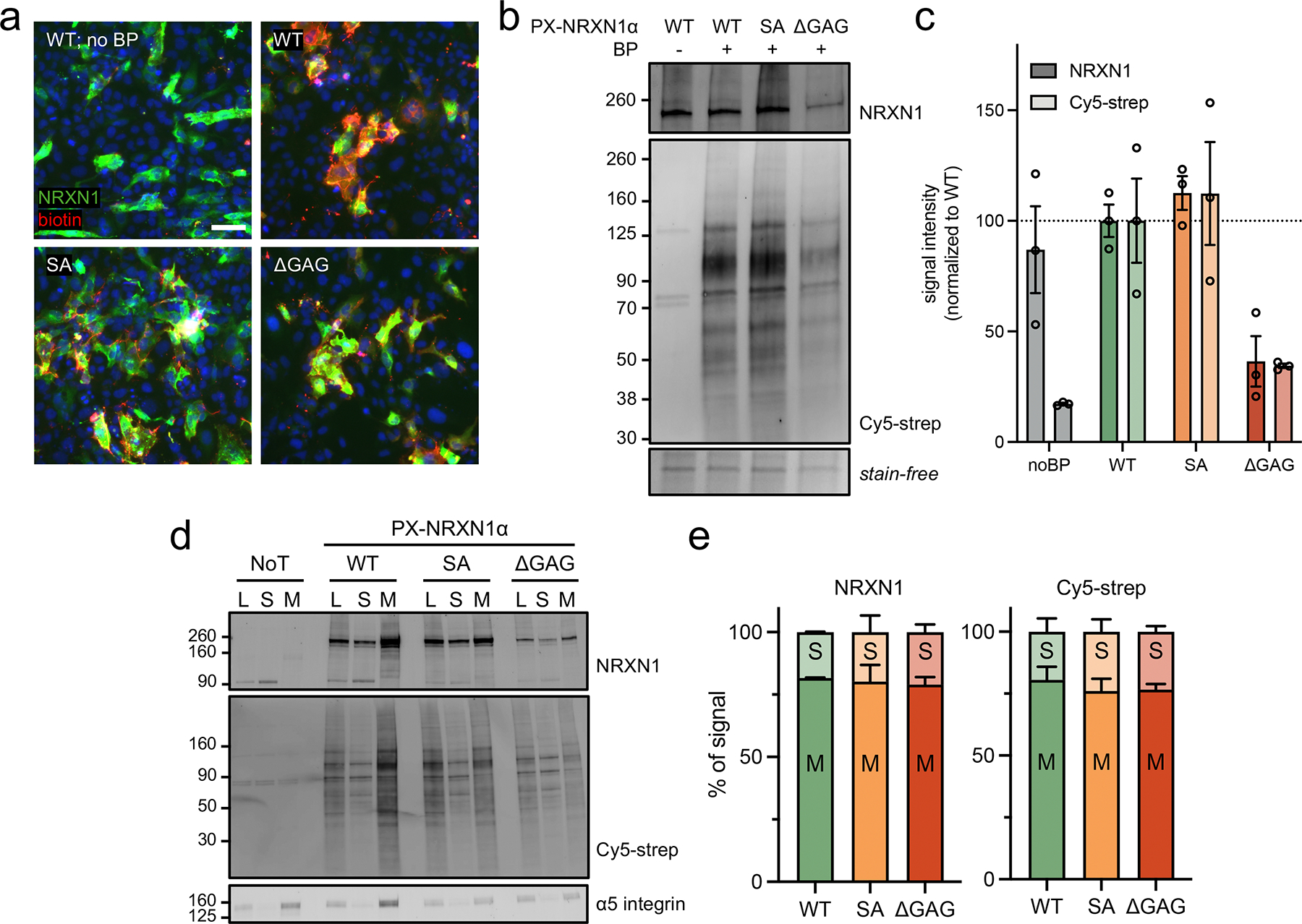
PX-NRXN1α for proximity labeling in SVG p12 cells. (a) Immunofluorescence microscopy demonstrates BP-dependent biotinylation (red, Cy5-streptavidin) and robust NRXN1α expression (green). Scale bar = 75 μm. (b, c) Western blot analysis and quantification of PX-NRXN1α expression and biotinylation intensity of cell lysates following proximity tagging. (d) Crude membrane separation by ultracentrifugation reveals that most biotinylated proteins present in lysate (L) are localized to membrane (M), not soluble (S) fractions. (e) Quantification of PX-NRXN1α expression (left) and biotin signal (right) distribution in membrane and soluble fractions. Data are representative of at least two biological replicates. One-way analysis of variance (ANOVA). Bar graphs represent means, and error bars represent SEM. NoT = non-transfected cells.

To determine whether our fusion constructs could mediate proximity labeling, SVG p12 cells were transiently transfected for 48 h before inducing labeling. Cells were incubated with biotin phenol (BP) before the addition of H_2_O_2_ to catalyze the generation of biotin-phenoxyl radicals that label electron-rich residues in proximal biomolecules ( < 20 nm, Figure 1b). This reaction is quenched before cells are processed for biotin detection or enrichment. Initial immunofluorescence microscopy demonstrated the ability of PX-NRXN1α constructs to mediate proximity labeling, dependent upon incubation with BP and PX-NRXN1α transfection (Figure 2a, Supporting Information S1: Figure S1a,b). By microscopy, PX-NRXN1α^WT^ appeared to result in more biotinylation than the SA or ΔGAG mutants, although protein expression appeared consistent in this qualitative format (Figure 2a, Supporting Information S1: Figure S2a). For a semi-quantitative evaluation of the impact of glycosylation on the interactome of NRXN1α, western blot analysis was performed on whole cell lysates after proximity labeling. The negative control for these experiments consisted of cells overexpressing PX-NRXN1α^WT^ that underwent the proximity labeling protocol except for BP incubation. Consistent with our microscopy data, the extent of biotinylation appears correlated to the number of GAG sites present (WT > SA > ΔGAG, Figure 2b). While we note that PX-NRXN1α^ΔGAG^ appears to be less expressed than other constructs, by normalization of total biotin signals to NRXN1, we confirm that the reduction in signal is not due to a decrease in total protein expression (Supporting Information S1: Figure S2c). Encouragingly, biotin intensity from PX-NRXN1α^ΔGAG^ lysates was approximately 60% lower than PX-NRXN1α^WT^, suggesting GAGs may play a significant role in the NRXN1α interactome (Figure 2c).

While we expected the presence and valency of GAG chains to influence the NRXN1α interactome, we sought to confirm that the changes seen were not due to differences in cellular localization. As NRXN1α is typically highly glycosylated, we postulated that the loss of one or more GAG sites in PX-NRXN1α could impact its folding and trafficking to the plasma membrane, thereby affecting the efficiency of proximity labeling. Importantly, only the GAG sites were mutated in this study, and all predicted N-glycosylation sites were kept intact. Thus, we performed ultracentrifugation on cell lysates after proximity labeling to yield discrete soluble and membrane fractions. Using the marker α5 integrin to confirm the separation of membrane-bound and cytosolic proteins, we observed that the majority of our PX-NRXN1α construct is localized to the membrane regardless of GAG site mutations (Figure 2d, Supporting Information S1: Figure S2d). Although some NRXN1α was detected in soluble fractions, the proportion was constant across our constructs (Figure 2e). In agreement with the majority of our APEX2 proteins localizing to membranes, we observed similar trends in biotinylation upon blotting with streptavidin (Figure 2e). This observation suggests that while PX-NRXN1α^ΔGAG^ is expressed at lower levels than our other constructs, the observed decreased biotinylation is not due to differential cellular localization. We cannot, however, rule out degradation of our GAG-null variant, which could explain the decreased expression observed.

To further confirm a role for GAGs in modulating its interactome, we pre-incubated PX-NRXN1α^WT^-transfected cells with heparin, a soluble, highly sulfated version of HS [42]. We hypothesized that the addition of heparin could disrupt and outcompete GAG-mediated interactions due to its higher sulfation and charge density [42]. It is widely known that PGs can form dynamic ternary complexes through their GAG chains [43, 44]. As such, we performed dose competition experiments with heparin to determine the optimal concentration of heparin to disrupt, rather than promote, interactions [45]. Using PX-NRXN1α^WT^ expressing cells, we observed only a slight decrease in biotin signal with 5 and 20 μg/mL heparin by western blot analysis (Supporting Information S1: Figure S2e–f). The maximal reduction in biotin signal for heparin was only 20% compared to 50% with PX-NRXN1α^ΔGAG^, suggesting minimal competition (Figure 1c, Supporting Information S1: Figure S2c,f). Further, the efficacy was variable between biological replicates, perhaps attesting to the dynamic nature of heparin interactions with the glycocalyx.

### 2.3 | Identification of the Astrocytic NRXN1α Interactome by MS-Based Proteomics

**FIGURE 3 |:**
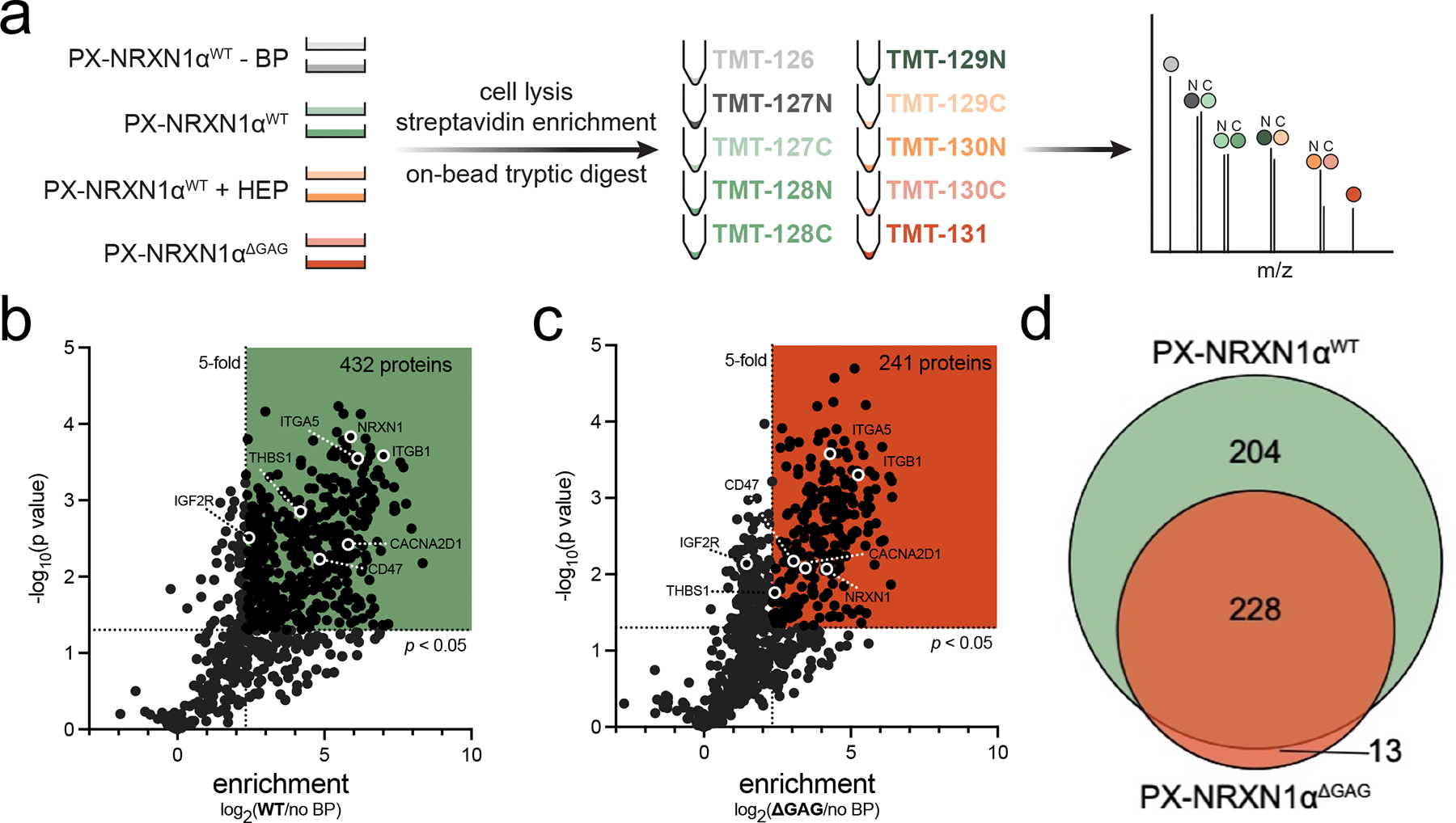
Identification and analysis of the PX-NRXN1α interactome in SVG p12 cells. (a) Workflow for capturing and digesting biotinylated proteins before labeling of peptides with isobaric TMT tags for subsequent mass spectrometry detection. (b, c) Volcano plots of proteins significantly enriched (*p* < 0.05, TMT ratio of PX-NRXN1α^WT/ΔGAG^/PX-NRXN1α^WT^ – BP ≥ 5) by (b) PX-NRXN1α^WT^ (green) and (c) PX-NRXN1α^ΔGAG^ (red). (d) Venn diagram comparing the number of significantly enriched proteins identified by WT and ΔGAG constructs.

To identify the putative NRXN1α interactors, SVG p12 cells were transiently transfected with PX-NRXN1α constructs for 48 h before being subjected to proximity labeling. PX-NRXN1α^WT^ and PX-NRXN1α^ΔGAG^ were utilized for proteomics experiments to maximize potential contrasting observations between wild type and GAG-null protein, to permit identification of GAG-mediated interactions. Cells were subsequently lysed and biotinylated proteins were enriched with streptavidin beads before on-bead tryptic digestion (Figure 3a). The resulting peptides underwent labeling with tandem mass tags (TMT) to chemically tag peptides from each cell treatment with unique isobaric tags distinguishable at the MS3 level [46] (Figure 3a). This allows for reliable quantitative analysis of multiplexed samples in a single injection.

A total of 568 proteins were identified across at least two independent proteomics experiments, each of which contained two biological replicates (Supporting Information S1: Figure S3a–c). These proteins were detected with at least two unique peptides and were significantly (*p* < 0.05) and highly enriched (TMT ratio ≥ 5) by PX-NRXN1α^WT^ over cells treated with PX-NRXN1α^WT^ without BP incubation. The raw TMT values across two biological replicates were normalized to levels of the endogenously biotinylated protein PCCA [37], resulting in 432 high-confidence targets enriched by our WT construct over controls (Figure 3b, Supporting Information S1: Figure Table S1,S3). Gene ontology analysis revelas that the most highly enriched cellular components were membrane associated (Supporting Information S1: Figure S4). These putative interactors include several proteins known to bind NRXNs, including CACNA2D1 [47] and THBS1 [48] (Table 1). In agreement with our observations by western blot analysis (Figure 2b,c), compared to our WT construct, substantially fewer proteins were significantly enriched by PX-NRXN1α^ΔGAG^ over control (241 vs. 432; Figure 3c). Of the 445 total proteins enriched by either construct, 228 (51%) were shared between WT and GAG null PX-NRXN1α (Figure 3d). Among these are proteins that have been previously reported to interact with GAGs, including multiple integrins (Table 1).

The inclusion of a heparin competition condition (5 μg/mL) in our proteomics analysis allowed us to compare the extent of interactome changes upon treatment of WT-expressing cells with soluble heparin and the GAG-null protein mutant (Figure 3a). Consistent with our western blot analysis, wherein ΔGAG had a much larger reduction in signal than heparin treatment (Figure 2c, Supporting Information S1: Figure S2c,f), pretreatment with heparin resulted in only 18 proteins being competed (Supporting Information S1: Figure S3d and Table S3). Relaxing the cut-off to 1.5-fold (TMT ratio of PX-NRXN1α^WT^/PX-NRXN1α^WT^ + heparin ≥ 1.5) only increased the protein count to 80 (Supporting Information S1: Figure S3d). These data agree with our previous observations (Figure 2c) and suggest that a glycosite mutant may be a better strategy for observing GAG-dependent interactions with a bait glycoprotein than the addition of a soluble competitor.

### 2.4 | Validation of GAG-Mediated PX-NRXN1α Interactions

**FIGURE 4 |:**
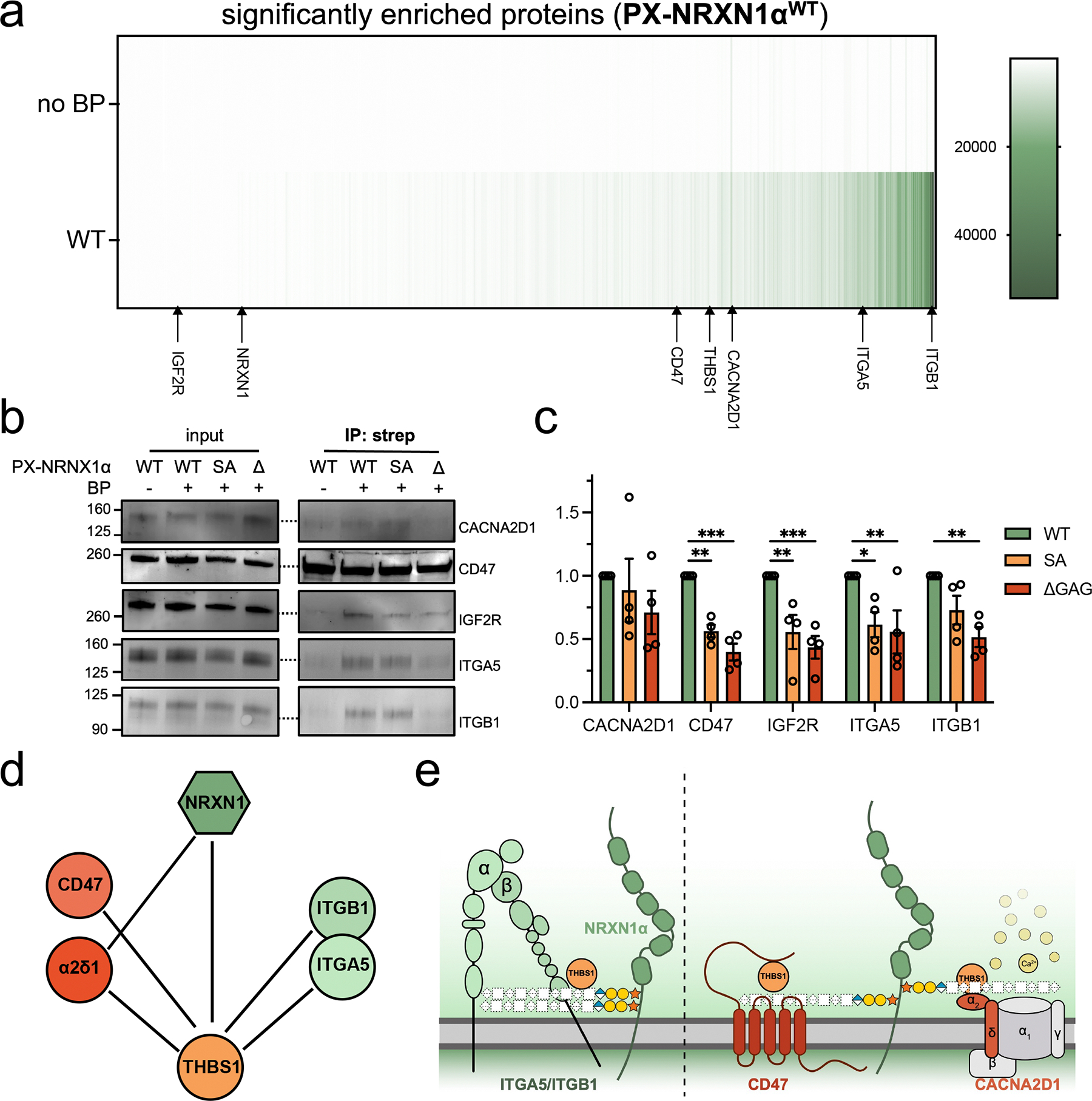
Some PX-NRXN1α interactions are GAG-mediated. (a) Heatmap depicting the TMT values for the 432 proteins significantly enriched by PX-NRXN1α^WT^. (b) Western blot analysis from streptavidin-enriched lysates probed for the differentially enriched proteins CACNA2D1, CD47, IGF2R, ITGA5, and ITGB1. (c) Quantification of western blot analysis. Data are representative of four biological replicates. One-way analysis of variance (ANOVA). Bar graphs represent means, and error bars represent SEM.

We then sought to validate the differentially enriched proteins from our generated PX-NRXN1α interactome data set. A heatmap depicting the raw TMT values of the 432 proteins significantly enriched by PX-NRXN1α^WT^ (TMT ratio of PX-NRXN1α^WT^/PX-NRXN1α^WT^ without BP ≥ 5) demonstrated that proteins differentially enriched by PX-NRXN1α^ΔGAG^ were observed in both low and high abundance proteins (Figure 4a). Among the statistically significant proteins are several previously identified NRXN1 interactors, including CACNA2D1 [47, 60] and THBS1 [61], and astrocyte resident, HS-interacting proteins that have been suggested as potential NRXN-binding proteins, including ephrins [62] and integrins [63] (Figure 4a). As THBS1 has been previously shown to interact with the voltage-dependent calcium channel subunit α2δ–1 (CACNA2D1) [64], CD47 [65, 66], and NRXN1 [61], we postulate whether NRXN1α could be orchestrating thrombospondin interactions, which have well-established impacts on synapse development [48, 64, 67, 68]. Data mining of our putative NRXN1α interactors yielded additional THBS1 interactors, including integrin α5β1 (ITGA5, ITGB1 [69–71]) and CD47 [65, 66] (Figure 4a), which have not been previously reported to interact with neurexins. However, as known heparin/HS-binding proteins [57, 72] (Table 1), GAG-mediated binding to these integrins is likely.

To validate the identification of these proteins by LC-MS/MS proteomics and their differential enrichment, we performed streptavidin enrichment on lysates after proximity labeling. Consistently, we observed maximal intensity in our PX-NRXN1α^WT^ expressing cells for ITGA5, CACNA2D1, IGF2R, and ITGB1 (Figure 4b, Supporting Information S1: Figure S5). Of these proteins, only CACNA2D1 did not result in a significant reduction in enrichment by GAG mutants, suggesting the core protein may play a larger role in this interaction (Figure 4c). In agreement with our mass spectrometry data (Figure 3b,c), the detection of CD47 was lower upon enrichment of GAG mutant lysates compared to WT (Figure 4b,c). Similar trends were observed with both IGF2R and ITGA5. Uniquely, the enrichment of ITGB1 was only significantly decreased with the GAG-null PX-NRXN1α^ΔGAG^ mutant, not PX-NRXN1α^SA^, suggesting this interaction may require GAG valency (Figure 4c). These data confirm the changes in enrichment observed between PX-NRXN1α^WT^ and PX-NRXN1α^ΔGAG^ by mass spectrometry-based proteomics and further suggest a role for GAG chains in modulating the glial interactome of NRXN1α.

## 3 | Discussion

The structures of neurexins are highly regulated, from the inclusion of splice sites to the presence, composition, and valency of GAGs [19, 20]. To date, NRXN family proteins are the only PGs to have small protein interactors that control GAG attachment in a cell-type-dependent manner, including FAM19A and CA10 [73–75]. The existence of these mechanisms implies that the GAG state of NRXNs is tightly linked to their functions. Indeed, even within a mixed neuron-glia culture, previous reports have demonstrated that differential glycosylation occurs and suggest that NRXNs can be modified with both HS and CS chains, in agreement with our observations (Figure 1b).

We have utilized live cell proximity labeling in SVG p12 cells to further elucidate the structure and interactome of NRXN1α, the most commonly expressed astrocytic isoform [33]. Our protein constructs were generated using isoform α2, the longest transcript, which includes the recently characterized splice site 5 and thus two potential GAG attachment sites [20]. GAG valency is an important aspect of many PGs to function correctly [76] or interact with certain proteins [22]. We sought to determine if the presence of GAG chains on NRXN1α greatly influenced its interactome. Here, we observe that a GAG-null mutant of PX-NRXN1α resulted in a ~50% reduction of protein interactors (Figure 3). Consistent with our previous efforts to define glycan-mediated binding [35–37], we utilized heparin as a soluble competitor to our GAG-modified, membrane-bound protein. However, both western blot and proteomics analysis of labeled proteins demonstrated substantially less reduction in interactions by heparin than our ΔGAG mutant (Supporting Information S1: Figure S2e). Quantification of biotin signal by western blot analysis resulted in only ~20% maximal reduction by heparin (Supporting Information S1: Figure S2f) compared to 40%–60% by our PX-NRXN1α GAG mutants (Figure 2c). These data suggest that future studies on the interactome of engineered glycoproteins should compare both glycosite mutants and soluble competitors (i.e., lactose or heparin) to determine true glycan-mediated interactions.

Many canonical interactors were not detected in our proteomics data set, such as NLGNs, likely given that previous studies have focused on neuronal NRXNs. The current cell line used is astrocyte-like but likely resembles glial progenitor cells, underscoring the need for future studies to apply these powerful proximity labeling tools to other glia types that express neurexins, including mature astrocyte cultures. Moreover, many physiologically relevant interactions occur at cell-cell interfaces; thus, simplified systems consisting solely of glial cells can uncover *cis* interactions and complex formation, but represent only an initial step toward understanding more intricate structures such as astrocyte-synapse intercellular junctions. A recent study in *C. elegans* applied TurboID-fused NRXN1 pan-neuronally to characterize the intracellular interactome [77]. A number of the proteins highlighted in this manuscript have human orthologs that are detected in our proteomics, including pat-3 (ITGB1), pat-2 (ITGA5), dgn-1 (DAG1), and unc-52 (HSPG2) [77], adding confidence to our datasets (Figure 3, Supporting Information S1: Table S1). Indeed, BioMart analysis of human homologs revealed that 155 proteins identified in *C. elegans* were detected as PX-NRXX1α interactors in human fetal astroglial cells, including lec-1 (LGALS1, Supporting Information S1: Figure S4). Further, the characterization of NRXNs as HS-modified PGs opens their potential interacting proteins to several heparin-binding proteins, including ephrins and integrins, which were not only abundant in our datasets but showed reduced enrichment by our GAG-null mutant PX-NRXN1^ΔGAG^, suggesting glycan-dependent interactions (Figure 3f). Two integrins robustly detected in our data set are ITGB1, which is widely expressed during neuroectoderm differentiation and upregulated in glial monoculture [78], and ITGA5 which has been linked to maintenance of synaptic integrity [79].

While we define the interactome of both PX-NRXN1α^WT^ and a GAG-null mutant, differences in their expression levels prohibit direct comparisons of protein enrichment between these conditions (Figure 2b–d). We have previously published a modular platform for the expression and cell surface display of engineered PGs [22]. Using this strategy, which we have further shown to apply to APEX2 fusion proteins [22], we envision the display of exogenous PX-NRXN1α in controlled concentrations for equal display of His-tagged GAG variants at the cell surface through lipid anchors [80]. A limitation of this strategy, however, is the resulting lack of transmembrane and intracellular domains, which have been demonstrated to have roles in many protein–protein interactions in NRXN1 [77].

## 4 | Materials and Methods

### 4.1 | Plasmid Preparation and Mutagenesis

DNA encoding APEX2 fused to NRXN1 isoform α2 (NM_001135659.3) was subcloned into the pcDNA3.1(+) vector for mammalian expression by GenScript. In these plasmids, the signal peptide sequence of NRXN1α (MGTALLQRGGCFLLCLSLLLLG CWAELGSG) was moved to the N-terminus, followed by a GSSGSS linker, APEX2, GSSGSS linker, a TEV cleavage site, followed by the remainder of NRXN1 isoform α2 and C-terminal FLAG. PX-NRXN1α^SA^ was similarly prepared by GenScript, mutating S1425A. PX-NRXN1α^ΔGAG^ was created from PX-NRXN1α^SA^ using Q5 Site-Directed Mutagenesis Kit (New England Biolabs, #E0554). Whole plasmid sequencing was performed on the resulting transformants by PlasmidSaurus.

**Table:** PX-NRXN1α amino acid sequences. Mutated sites.

PX-NRXN1α^WT^	MGTALLQRGGCFLLCLSLLLLGCWAELGSGGSSGSSGKSYPTVSADYQDAVEKAKKKLRGFIAEKRCAPLMLRLAFHSAGTFDKGTKTGGPFGTIKHPAELAHSANNGLDIAVRLLEPLKAEFPILSYADFYQLAGVVAVEVTGGPKVPFHPGREDKPEPPPEGRLPDPTKGSDHLRDVFGKAMGLTDQDIVALSGGHTIGAAHKERSGFEGPWTSNPLIFDNSYFTELLSGEKEGLLQLPSDKALLSDPVFRPLVDKYAADEDAFFADYAEAHQKLSELGFADAGSSGSSENLYFQSLEFPGAEGQWTRFPKWNACCESEMSFQLKTRSARGLVLYFDDEGFCDFLELILTRGGRLQLSFSIFCAEPATLLADTPVNDGAWHSVRIRRQFRNTTLFIDQVEAKWVEVKSKRRDMTVFSGLFVGGLPPELRAAALKLTLASVREREPFKGWIRDVRVNSSQVLPVDSGEVKLDDEPPNSGGGSPCEAGEEGEGGVCLNGGVCSVVDDQAVCDCSRTGFRGKDCSQEIKFGLQCVLPVLLHDNDQGKYCCINTAKPLTEKDNNVEGLAHLMMGDQGKSKGKEEYIATFKGSEYFCYDLSQNPIQSSSDEITLSFKTLQRNGLMLHTGKSADYVNLALKNGAVSLVINLGSGAFEALVEPVNGKFNDNAWHDVKVTRNLRQHSGIGHAMVNKLHCSVTISVDGILTTTGYTQEDYTMLGSDDFFYVGGSPSTADLPGSPVSNNFMGCLKEVVYKNNDVRLELSRLAKQGDPKMKIHGVVAFKCENVATLDPITFETPESFISLPKWNAKKTGSISFDFRTTEPNGLILFSHGKPRHQKDAKHPQMIKVDFFAIEMLDGHLYLLLDMGSGTIKIKALLKKVNDGEWYHVDFQRDGRSGTISVNTLRTPYTAPGESEILDLDDELYLGGLPENKAGLVFPTEVWTALLNYGYVGCIRDLFIDGQSKDIRQMAEVQSTAGVKPSCSKETAKPCLSNPCKNNGMCRDGWNRYVCDCSGTGYLGRSCEREATVLSYDGSMFMKIQLPVVMHTEAEDVSLRFRSQRAYGILMATTSRDSADTLRLELDAGRVKLTVNLDCIRINCNSSKGPETLFAGYNLNDNEWHTVRVVRRGKSLKLTVDDQQAMTGQMAGDHTRLEFHNIETGIITERRYLSSVPSNFIGHLQSLTFNGMAYIDLCKNGDIDYCELNARFGFRNIIADPVTFKTKSSYVALATLQAYTSMHLFFQFKTTSLDGLILYNSGDGNDFIVVELVKGYLHYVFDLGNGANLIKGSSNKPLNDNQWHNVMISRDTSNLHTVKIDTKITTQITAGARNLDLKSDLYIGGVAKETYKSLPKLVHAKEGFQGCLASVDLNGRLPDLISDALFCNGQIERGCEGPSTTCQEDSCSNQGVCLQQWDGFSCDCSMTSFSGPLCNDPGTTYIFSKGGGQITYKWPPNDRPSTRADRLAIGFSTVQKEAVLVRVDSSSGLGDYLELHIHQGKIGVKFNVGTDDIAIEESNAIINDGKYHVVRFTRSGGNATLQVDSWPVIERYPAGNNDNERLAIARQRIPYRLGRVVDEWLLDKGRQLTIFNSQATIIIGGKEQGQPFQGQLSGLYYNGLKVLNMAAENDANIAIVGNVRLVGEVPSSMTTESTATAMQSEMSTSIMETTTTLATSTARRGKPPTKEPISQTTDDILVASAECPSDDEDIDPCEPSSGGLANPTRAGGREPYPGSAEVIRESSSTTGMVVGIVAAAALCILILLYAMYKYRNRDEGSYHVDESRNYISNSAQSNGAVVKEKQPSSAKSSNKNKKNKDKEYYVDYKDDDDK
PX-NRXN1α^SA^ S1425A	MGTALLQRGGCFLLCLSLLLLGCWAELGSGGSSGSSGKSYPTVSADYQDAVEKAKKKLRGFIAEKRCAPLMLRLAFHSAGTFDKGTKTGGPFGTIKHPAELAHSANNGLDIAVRLLEPLKAEFPILSYADFYQLAGVVAVEVTGGPKVPFHPGREDKPEPPPEGRLPDPTKGSDHLRDVFGKAMGLTDQDIVALSGGHTIGAAHKERSGFEGPWTSNPLIFDNSYFTELLSGEKEGLLQLPSDKALLSDPVFRPLVDKYAADEDAFFADYAEAHQKLSELGFADAGSSGSSENLYFQSLEFPGAEGQWTRFPKWNACCESEMSFQLKTRSARGLVLYFDDEGFCDFLELILTRGGRLQLSFSIFCAEPATLLADTPVNDGAWHSVRIRRQFRNTTLFIDQVEAKWVEVKSKRRDMTVFSGLFVGGLPPELRAAALKLTLASVREREPFKGWIRDVRVNSSQVLPVDSGEVKLDDEPPNSGGGSPCEAGEEGEGGVCLNGGVCSVVDDQAVCDCSRTGFRGKDCSQEIKFGLQCVLPVLLHDNDQGKYCCINTAKPLTEKDNNVEGLAHLMMGDQGKSKGKEEYIATFKGSEYFCYDLSQNPIQSSSDEITLSFKTLQRNGLMLHTGKSADYVNLALKNGAVSLVINLGSGAFEALVEPVNGKFNDNAWHDVKVTRNLRQHSGIGHAMVNKLHCSVTISVDGILTTTGYTQEDYTMLGSDDFFYVGGSPSTADLPGSPVSNNFMGCLKEVVYKNNDVRLELSRLAKQGDPKMKIHGVVAFKCENVATLDPITFETPESFISLPKWNAKKTGSISFDFRTTEPNGLILFSHGKPRHQKDAKHPQMIKVDFFAIEMLDGHLYLLLDMGSGTIKIKALLKKVNDGEWYHVDFQRDGRSGTISVNTLRTPYTAPGESEILDLDDELYLGGLPENKAGLVFPTEVWTALLNYGYVGCIRDLFIDGQSKDIRQMAEVQSTAGVKPSCSKETAKPCLSNPCKNNGMCRDGWNRYVCDCSGTGYLGRSCEREATVLSYDGSMFMKIQLPVVMHTEAEDVSLRFRSQRAYGILMATTSRDSADTLRLELDAGRVKLTVNLDCIRINCNSSKGPETLFAGYNLNDNEWHTVRVVRRGKSLKLTVDDQQAMTGQMAGDHTRLEFHNIETGIITERRYLSSVPSNFIGHLQSLTFNGMAYIDLCKNGDIDYCELNARFGFRNIIADPVTFKTKSSYVALATLQAYTSMHLFFQFKTTSLDGLILYNSGDGNDFIVVELVKGYLHYVFDLGNGANLIKGSSNKPLNDNQWHNVMISRDTSNLHTVKIDTKITTQITAGARNLDLKSDLYIGGVAKETYKSLPKLVHAKEGFQGCLASVDLNGRLPDLISDALFCNGQIERGCEGPSTTCQEDSCSNQGVCLQQWDGFSCDCSMTSFSGPLCNDPGTTYIFSKGGGQITYKWPPNDRPSTRADRLAIGFSTVQKEAVLVRVDSSSGLGDYLELHIHQGKIGVKFNVGTDDIAIEESNAIINDGKYHVVRFTRSGGNATLQVDSWPVIERYPAGNNDNERLAIARQRIPYRLGRVVDEWLLDKGRQLTIFNSQATIIIGGKEQGQPFQGQLSGLYYNGLKVLNMAAENDANIAIVGNVRLVGEVPSSMTTESTATAMQSEMSTSIMETTTTLATSTARRGKPPTKEPISQTTDDILVAAAECPSDDEDIDPCEPSSGGLANPTRAGGREPYPGSAEVIRESSSTTGMVVGIVAAAALCILILLYAMYKYRNRDEGSYHVDESRNYISNSAQSNGAVVKEKQPSSAKSSNKNKKNKDKEYYVDYKDDDDK
PX-NRXN1α^ΔGAG^ S1425A/S1441A/S1442A	MGTALLQRGGCFLLCLSLLLLGCWAELGSGGSSGSSGKSYPTVSADYQDAVEKAKKKLRGFIAEKRCAPLMLRLAFHSAGTFDKGTKTGGPFGTIKHPAELAHSANNGLDIAVRLLEPLKAEFPILSYADFYQLAGVVAVEVTGGPKVPFHPGREDKPEPPPEGRLPDPTKGSDHLRDVFGKAMGLTDQDIVALSGGHTIGAAHKERSGFEGPWTSNPLIFDNSYFTELLSGEKEGLLQLPSDKALLSDPVFRPLVDKYAADEDAFFADYAEAHQKLSELGFADAGSSGSSENLYFQSLEFPGAEGQWTRFPKWNACCESEMSFQLKTRSARGLVLYFDDEGFCDFLELILTRGGRLQLSFSIFCAEPATLLADTPVNDGAWHSVRIRRQFRNTTLFIDQVEAKWVEVKSKRRDMTVFSGLFVGGLPPELRAAALKLTLASVREREPFKGWIRDVRVNSSQVLPVDSGEVKLDDEPPNSGGGSPCEAGEEGEGGVCLNGGVCSVVDDQAVCDCSRTGFRGKDCSQEIKFGLQCVLPVLLHDNDQGKYCCINTAKPLTEKDNNVEGLAHLMMGDQGKSKGKEEYIATFKGSEYFCYDLSQNPIQSSSDEITLSFKTLQRNGLMLHTGKSADYVNLALKNGAVSLVINLGSGAFEALVEPVNGKFNDNAWHDVKVTRNLRQHSGIGHAMVNKLHCSVTISVDGILTTTGYTQEDYTMLGSDDFFYVGGSPSTADLPGSPVSNNFMGCLKEVVYKNNDVRLELSRLAKQGDPKMKIHGVVAFKCENVATLDPITFETPESFISLPKWNAKKTGSISFDFRTTEPNGLILFSHGKPRHQKDAKHPQMIKVDFFAIEMLDGHLYLLLDMGSGTIKIKALLKKVNDGEWYHVDFQRDGRSGTISVNTLRTPYTAPGESEILDLDDELYLGGLPENKAGLVFPTEVWTALLNYGYVGCIRDLFIDGQSKDIRQMAEVQSTAGVKPSCSKETAKPCLSNPCKNNGMCRDGWNRYVCDCSGTGYLGRSCEREATVLSYDGSMFMKIQLPVVMHTEAEDVSLRFRSQRAYGILMATTSRDSADTLRLELDAGRVKLTVNLDCIRINCNSSKGPETLFAGYNLNDNEWHTVRVVRRGKSLKLTVDDQQAMTGQMAGDHTRLEFHNIETGIITERRYLSSVPSNFIGHLQSLTFNGMAYIDLCKNGDIDYCELNARFGFRNIIADPVTFKTKSSYVALATLQAYTSMHLFFQFKTTSLDGLILYNSGDGNDFIVVELVKGYLHYVFDLGNGANLIKGSSNKPLNDNQWHNVMISRDTSNLHTVKIDTKITTQITAGARNLDLKSDLYIGGVAKETYKSLPKLVHAKEGFQGCLASVDLNGRLPDLISDALFCNGQIERGCEGPSTTCQEDSCSNQGVCLQQWDGFSCDCSMTSFSGPLCNDPGTTYIFSKGGGQITYKWPPNDRPSTRADRLAIGFSTVQKEAVLVRVDSSSGLGDYLELHIHQGKIGVKFNVGTDDIAIEESNAIINDGKYHVVRFTRSGGNATLQVDSWPVIERYPAGNNDNERLAIARQRIPYRLGRVVDEWLLDKGRQLTIFNSQATIIIGGKEQGQPFQGQLSGLYYNGLKVLNMAAENDANIAIVGNVRLVGEVPSSMTTESTATAMQSEMSTSIMETTTTLATSTARRGKPPTKEPISQTTDDILVAAAECPSDDEDIDPCEPAAGGLANPTRAGGREPYPGSAEVIRESSSTTGMVVGIVAAAALCILILLYAMYKYRNRDEGSYHVDESRNYISNSAQSNGAVVKEKQPSSAKSSNKNKKNKDKEYYVDYKDDDDK

### 4.2 | Cell Culture

SVG p12 cells (CRL-8621) were purchased from ATCC and cultured in EMEM (ATCC, #30–2003) supplemented with 10% FBS (Omega Scientific #FB-01) and 1% penicillin–streptomycin (Corning, #30–002-CI). Cells were seeded at 2 × 10^6^ cells in a 10 cm^2^ plate for proteomics, 6 × 10^5^ in a 10 cm^2^ plate for western blot analysis, and 5 × 10^4^ per well in a 24-well plate for immunofluorescence microscopy.

### 4.3 | PX-NRXN1α Expression

Twenty-four hours after seeding, SVG p12 cells were transfected using FuGENE HD (Promega, #E2311) following the manufacturer’s instructions. In short, plasmid encoding PX-NRXN1α was added to Opti-MEM (Gibco, #31985070) media at a ratio of 1 μg per 50 μL media, and a FuGENE HD:DNA ratio of 3:1. Transfection mixtures were incubated for 15 min at room temp before drop-wise addition to cells. Cells were cultured for 48 h before analysis.

### 4.4 | Live Cell Proximity Labeling

For heparin competition conditions, cells were pre-incubated with heparin (Iduron, #HEP001) in serum-free EMEM for 20 min at 37°C. Cells were then incubated with BP (500 μM in serum-free EMEM) for 20 min at 37°C before addition of H_2_O_2_ (1 mM, 1 min, RT). Labeling was quenched by three washes of quenching solution (5 mM Trolox, 10 mM sodium ascorbate, and 10 mM sodium azide in 1X PBS) before downstream analysis.

### 4.5 | Immunofluorescence

After proximity labeling, cells were fixed in 4% paraformaldehyde (10 min, RT) followed by two washes with PBS. Cells were incubated with anti-NRXN1α (Sigma-Aldrich, #ABN161-I, 1:1000) in 3% BSA/PBST for 1 h at RT before three PBS washes and incubation with streptavidin-Cy5 (Southern Biotechnology, #7105–15, 1:1000) and anti-rabbit IgG conjugated AlexaFluor-555 (Invitrogen, #A-21428, 1:3000) for 1 h at room temp. Lastly, cells were washed three times with PBS and incubated with 1 μg/mL Hoechst (20 min, RT). Cells were washed with PBS and imaged on an EVOS M5000 cell imaging system (Thermo Fisher Scientific).

### 4.6 | Western Blot Analysis

Cells were scraped and pelleted (600×*g*, 3 min) before lysis in 1X RIPA supplemented with Halt Protease Inhibitor Cocktail (Thermo Scientific, #78429) on ice for 15 min. Lysate was clarified by centrifugation (16,000×*g*, 15 min, 4°C). Supernatant was analyzed by DC Protein Assay (Bio-Rad). Whole cell lysate was normalized and prepared for SDS-PAGE analysis in Laemmli buffer (BioRad #1610747) with 10% 2-mercaptoethanol before being analyzed on 4%–15% gradient stain-free gels (BioRad). Proteins were transferred using a Trans-Blot Turbo System (BioRad) and rinsed in TBST before being blocked in 5% BSA/TBST (1 h, RT). Antibodies were incubated in 5% BSA/TBST (1 h, RT or 4°C, overnight) before, washed three times with TBST before incubation with secondary antibodies (1 h, RT). Blots were washed three times with TBST before imaging on a ChemiDoc MP system. Blots were subsequently stripped with Restore PLUS western blot stripping buffer (Thermo Scientific, 46430) for 10 min at RT.

### 4.7 | GAG Digestion

SVG p12 cells expressing PX-NRXN1α constructs were scraped and pelleted (600×*g*, 3 min) before lysis in 1X RIPA supplemented with Halt Protease Inhibitor Cocktail (Thermo Scientific, #78429) on ice for 15 min. Lysate was clarified by centrifugation (16,000×*g*, 15 min, 4°C) before quantification by DC Protein Assay (Bio-Rad). 20 μg of lysate was incubated with 0.1 U/mL chondroitinase ABC (Sigma, C2905–10UN) and 0.5 U/mL of heparinase I/II/III cocktail (in-house) at 37°C overnight. Laemmli buffer (BioRad #1610747) with 10% 2-mercaptoethanol was added before samples were boiled for 5 min and subsequently analyzed by SDS-PAGE as above.

### 4.8 | Ultracentrifugation

After proximity labeling, cells were pelleted (600×*g*, 3 min) before sonication in 100 μL cold 1X PBS with a Branson SFX250 sonicator (15 ms on, 40 ms off, 15% amplitude). Lysates were quantified by DC Protein Assay (Bio-Rad) and normalized, retaining 10% of total protein for input control. Equal volumes of the remaining lysates were subjected to ultracentrifugation in a Beckman-Coulter Optima MAX-XP centrifuge (100,000×*g*, 1 h, 4°C). The soluble supernatant was retained in a separate tube and the membrane fraction was resuspended in 6 M urea/PBS to improve solubility. Samples were then prepared for western blot analysis as above.

### 4.9 | Proteomics Sample Preparation

Proteomics experiments were performed with technical replicates of each condition. After proximity labeling, cells were pelleted (600 × *g*, 3 min) and resuspended in 200 μL 1X PBS before sonication with a Branson SFX250 sonicator (15 ms on, 40 ms off, 15% amplitude). Protein concentrations were analyzed using the DC Protein Assay (Bio-Rad), with 5 μg of whole cell lysate retained for western blot analysis. At least 500 μg of lysate was used for each condition, being added to a 15 mL tube with 2 mL of ice cold methanol before precipitation overnight at −20°C. The following day, the samples were vortexed and pelleted by centrifugation (5000 rpm, 10 min, 4°C). The protein pellet was washed with 2 mL of cold methanol by sonication and pelleted. The resulting pellet was dissolved in 500 μL of freshly prepared proteomics-grade urea (6 M in PBS) with 10 μL of 10% SDS. Disulfide bonds were reduced by adding 50 μL of freshly prepared 1:1 solution of TCEP (200 mM in PBS) and K_2_CO_3_ (600 mM in PBS) for 30 min at 37°C on a shaker. Proteins were alkylated by the addition of 70 μL of iodoacetamide (400 mM in PBS) and incubation for 30 min (RT, dark). To each solution, 130 μL of 10% SDS in PBS was added, diluted with PBS (5.5 mL) before incubation with streptavidin-agarose beads (100 μL of 50% slurry; ThermoScientific, #20353) for 1.5 h at RT while rotating. The streptavidin beads were pelleted by centrifugation (2000 rpm, 2 min, 4°C) and sequentially washed once each with 5 mL of the following: 0.2% SDS in PBS, PBS, and 100 mM TEAB [triethylammonium bicarbonate], pH 8.5. The beads were transferred into LoBind microcentrifuge tubes (Eppendorf; #022431081), and bound proteins were digested overnight at 37°C in 200 μL of trypsin solution containing sequence-grade trypsin (2 mg; Promega #V5111) supplemented with calcium chloride (1 mM in H_2_O). The beads were pelleted by centrifugation, and the supernatant containing the digested peptides was transferred to fresh LoBind tubes. Beads were washed with 100 μL TEAB and added to the LoBind tube (Thermo Scientific; #90406). In general, for each sample, 8 μL of the 20 μg/μL stock of TMT reagent was added along with dry MS-grade acetonitrile to achieve a final acetonitrile concentration of ~30% (vol/vol), followed by incubation at 37°C for 1 h. The reaction was quenched with 6 μL of hydroxylamine and incubated for 15 min before acidification with 4 μL of formic acid. The TMT-labeled samples were dried via vacuum centrifugation, and the samples were combined by redissolving one sample with 300 μL of buffer A (5% MeCN in H_2_O, 0.1% formic acid) and transferring the solution into each sample tube until all samples were redissolved (final volume ~300 μL). The fully combined sample was desalted using two C-18 columns (Thermo Scientific; #89870) according to the manufacturer’s instructions. The combined sample was dried via vacuum centrifugation and stored at −80°C until ready for injection.

### 4.10 | Liquid Chromatography Mass Spectrometry (LC/MS) Analysis

TMT-labeled peptides were resuspended in buffer A (20 μL, 0.1% formic acid in water) before LC-MS analysis. 3 μL of each sample was loaded onto an Acclaim PepMap 100 precolumn (75 μm × 2 mm) and eluted on an Acclaim PepMap RSLC analytical column (75 μm × 15 cm) using an UltiMate 3000 RSLCnano system (Thermo Fisher Scientific). Buffer A, prepared as above, and buffer B (0.1% formic acid in MeCN) were used in a 220 min gradient (flow rate 0.3 mL min, 35°C) of 2% buffer B for 10 min, an gradient to 30% buffer B over 192 min, 60% buffer B (5 min), 60%–95% buffer B for 1 min, hold at 95% buffer B (5 min), followed by descent to 2% buffer B (1 min) followed by re-equilibration at 2% buffer B (6 min).

### 4.11 | SPS-MS3 Method for Proteomics

Eluents from LC were analyzed with a Thermo Fisher Scientific Orbitrap Fusion Lumos mass spectrometer with a cycle time of 3 s and nano-LC electrospray ionization source applied voltage of 2.0 kV. MS1 spectra were recorded at a resolution of 120,000 with an automatic gain control (AGC) value of 1 × 10^6^ ions, maximum injection time of 50 ms (dynamic exclusion enabled, repeat count 1, duration 20 s). The scan range was specified from 375 to 1500 m/z. Peptides selected for MS2 analysis were isolated with the quadrupole (isolation window 1.6 m/z) and fragmented using collision-induced dissociation (CID 30%) with resultant fragments recorded in the ion trap (AGC 1.8 × 10^4^, maximum inject time 120 ms).

### 4.12 | Proteomics Analysis

Proteomics data were processed in Proteome Discoverer 3.2 (Thermo Scientific). Peptide sequences were determined by matching databases with the acquired fragmentation pattern by the SEQUEST HT algorithm. Precursor mass tolerance was set to 10 ppm and fragment ion mass tolerance to 0.6 Da. A single missed cleavage site of trypsin was allowed. Carbamidomethyl and TMT-10 plex were assigned as static modifications. Oxidation was used as a variable modification. Spectra were searched against the proteome database of *Homo sapiens* (83,374 sequences, UniProt, UP000005640) using a target false discovery rate of 1%. Proteins identified were filtered by at least two unique peptides before TMT values were obtained, with each data set containing two technical replicates of each experimental condition. Proteomics experiments were performed in two biological replicates. To identify significantly enriched proteins, proteomics runs for each cell line were normalized based on the detection of the endogenously biotinylated protein PCCA, to reduce variation between experiments [81]. Normalized TMT values were transformed by log2(x), and *p*-values were obtained by the *t*-test function in Prism 10 (GraphPad).

### 4.13 | Streptavidin Enrichment

After proximity labeling, cells were pelleted (600×*g*, 3 min) before lysis in 1X RIPA supplemented with Halt Protease Inhibitor Cocktail (Thermo Scientific, #78429) on ice for 15 min. Lysate was clarified by centrifugation (16,000×*g*, 15 min, 4°C). Supernatant was analyzed by DC Protein Assay (Bio-Rad) and normalized. 20 μg of whole cell lysate was retained as input, and equal quantities of protein were added to PBS-washed streptavidin-agarose beads (Thermo Scientific, #20353) for enrichment and rotated overnight at 4°C. Beads were washed twice with 1X PBS, once with 2M urea/PBS and a final 1X PBS. 2X Laemmli buffer with 10% 2-mercaptoethanol and 3 mM biotin-tyramide was added to the beads before boiling (95°C, 5 min) to elute enriched proteins for western blot analysis.

**Table:** Antibody list.

Protein	Manufacturer’s	Product #

NRXN1	Invitrogen	ABN161-I
ITGA5	Abcam	AB150361
ITGB1	Cell Signaling Technology	4706S
CACNA2D1	Invitrogen	MA3921
IGF2R	Cell Signaling Technology	14364S
CD47	Novus Biologicals	NBP2-31106
Streptavidin-Cy5	Southern Biotech	7100-15
Donkey antimouse AF555	Invitrogen	A31570
Goat anti-rabbit IgG AF647	Invitrogen	A32733

## 5 | Conclusions

We present the first extracellular interactome study of NRXN1 using proximity labeling that considers its status as a PG. Performed in a human astroglial cell line, our data set of 445 putative interactors includes several established NRXN1 interactors (DAG1, CACNA2D1, THBS1) and confirms interactions recently discovered by intracellular TurboID [77]. Included amongst our data set are numerous known HS-binding proteins previously hypothesized to interact with NRXN1 upon discovery of its GAG chains [19]. Indeed, ~50% of proteins identified by LC-MS/MS were differentially enriched by NRXN1α without GAG chains (Figures 3b,c and 4a).

Thus, our findings identify the possibility of targeting NRXN1 glycosylation to modulate interactions with many astrocytic proteins to understand further how structural GAG motifs can contribute to structure–function relationships. Neurexins and many of their ligands have been linked to synaptic transmission in circuits relevant to neuropsychiatric disorders [67]. Thus, our findings identify a potential axis for NRXN1 modulation of astrocyte–synapse interactions that influence synaptic function in neuropsychiatric disorders.

## Supplementary Material

Supporting Information

Table S1-S3 included as tabs

Additional supporting information can be found online in the Supporting Information section.

PX-NRNX1a SuppTables 20250722. PX-NRNX1a SI Resub 091625 MC.

## Figures and Tables

**TABLE 1 |:** Abbreviated list of PX-NRXN1^WT^ interactors identified by LC-MS/MS proteomics.

Gene name	Protein name	Known interactor?	Reported GAG binding?

CACNA2D1	Voltage-dependent calcium channel subunit α2δ–1	Yes [47]	Yes [49]
CALR	Calreticulin	No	Yes [50]
CALU	Calumenin	No	No
CD47	CD47/Integrin-associated protein (IAP)	No	PG [51]
CSPG4	Chondroitin sulfate proteoglycan 4/Neuron-glial antigen 2	No	PG [52]
DAG1	Dystroglycan	Yes [53]	No
EGFR	Epidermal growth factor receptor	No	Yes [54]
HSPG2	Heparan sulfate proteoglycan 2/Perlecan	No	PG [55]
IGF2R	Insulin-like growth factor 2 receptor	No	Yes [56]
ITGA5	Integrin alpha 5	No	Yes [57]
ITGB1	Integrin beta 1	No	Yes [57]
LGALS1	Galectin-1	No	Yes [58]
NECTIN2	Nectin2	No	No
NOTCH2	Notch2	No	No
NPTN	Neuroplastin	No	No
THBS1	Thrombospondin 1	Yes [48]	Yes [59]

Abbreviation: PG = proteoglycan/reported to have its own GAG chains.

## Data Availability

The mass spectrometry proteomics data have been deposited to the ProteomeXchange Consortium via the PRIDE [1] partner repository with the dataset identifier PXD069099 and 10.6019/PXD069099.
