# Supplementary material for: The Glycosaminoglycan-Dependent Interactome of Neurexin-1 in Human Fetal Glial Cells
Source: Proteoglycan Res. Author manuscript; Available in PMC 2025 Dec 17. (PMC12707391; doi:10.1002/pgr2.70039)
Supplement: Supporting Information [file NIHMS2117350-supplement-Supporting_Information.pdf]

The glycosaminoglycan-dependent interactome of neurexin-1 in human fetal glial cells

Meg Critcher,[1,] Han Wu,[1,] Yajing Lu,[1] Mia L. Huang[1*]

[1] Department of Chemistry, Scripps Research, 10550 N Torrey Pines Rd. La Jolla, CA 92037

* Corresponding author email: miahuang@scripps.edu

Table of contents:

Figure S1. Uncropped western blots for PX-NRXN1α characterization.

Figure S2. Optimization of proximity labeling for identification of GAG-mediated interactions.

Figure S3. Additional PX-NRNX1α proteomics analysis.

Figure S4. Gene Ontology analysis of proteomic hits in **Figure 3**.

Figure S5. Uncropped western blots for streptavidin enrichment

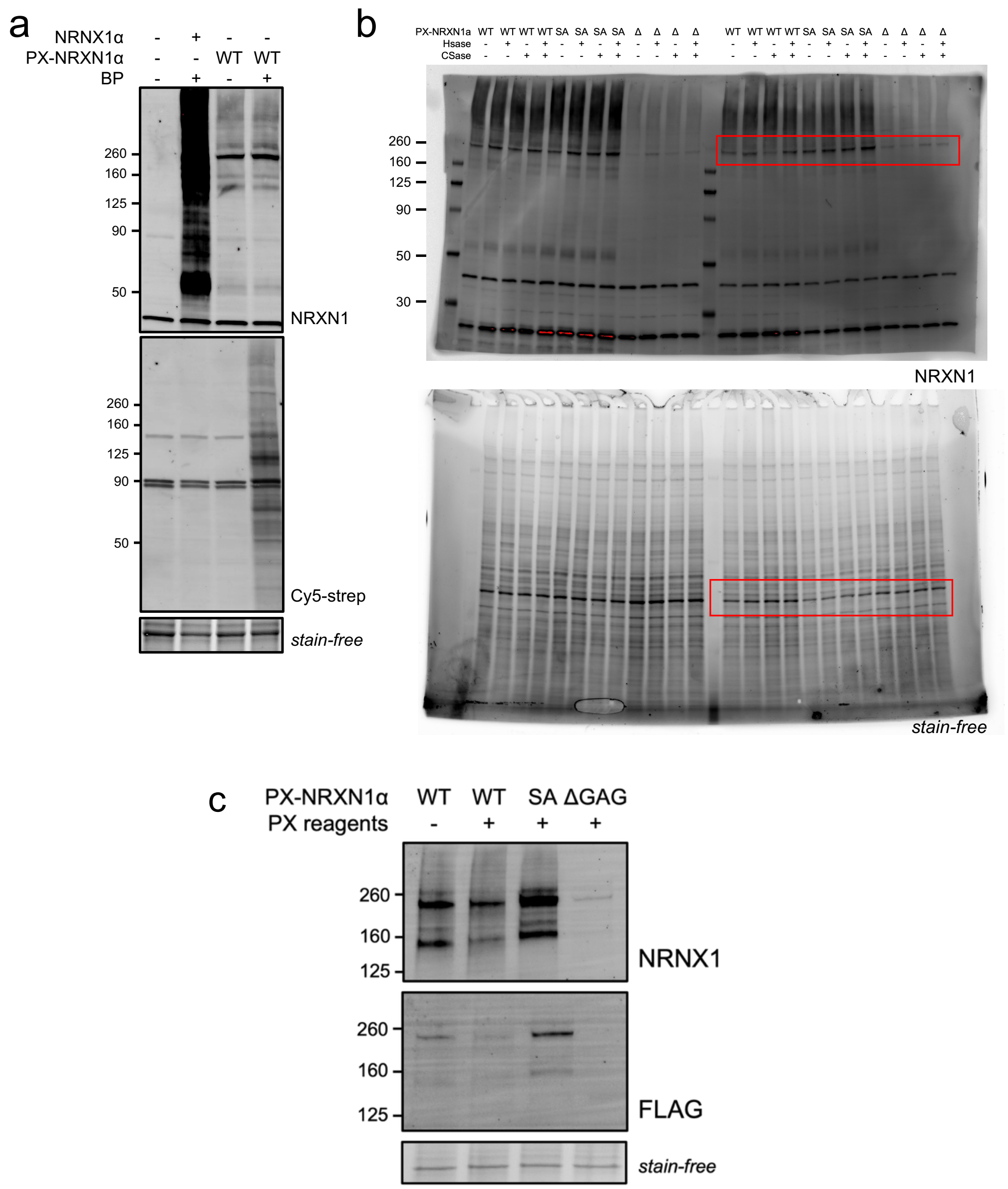

Supplemental Figure 1. Uncropped western blots for PX-NRXN1α characterization. (**a**) Western blot of SVG p12 cells expressing wild-type NRNX1α or PX-NRXN1α and subjected to proximity labeling. Lysates from non-transfected cell lysates display no observable NRXN1. Exclusion of biotin phenol (BP) during proximity labeling with PX-NRXN1α^{WT} results in detection of endogenously biotinylated proteins only. Expression of NRXN1α^{WT} is significantly higher than its APEX2-fused counterpart. (**b**) Figure 1c, areas used in main text highlighted in red. Top: NRNX1, bottom: stain-free gel. (**c**) Detection of protein expression via FLAG tag reveals reduced expression of ΔGAG compared to SA and WT constructs.

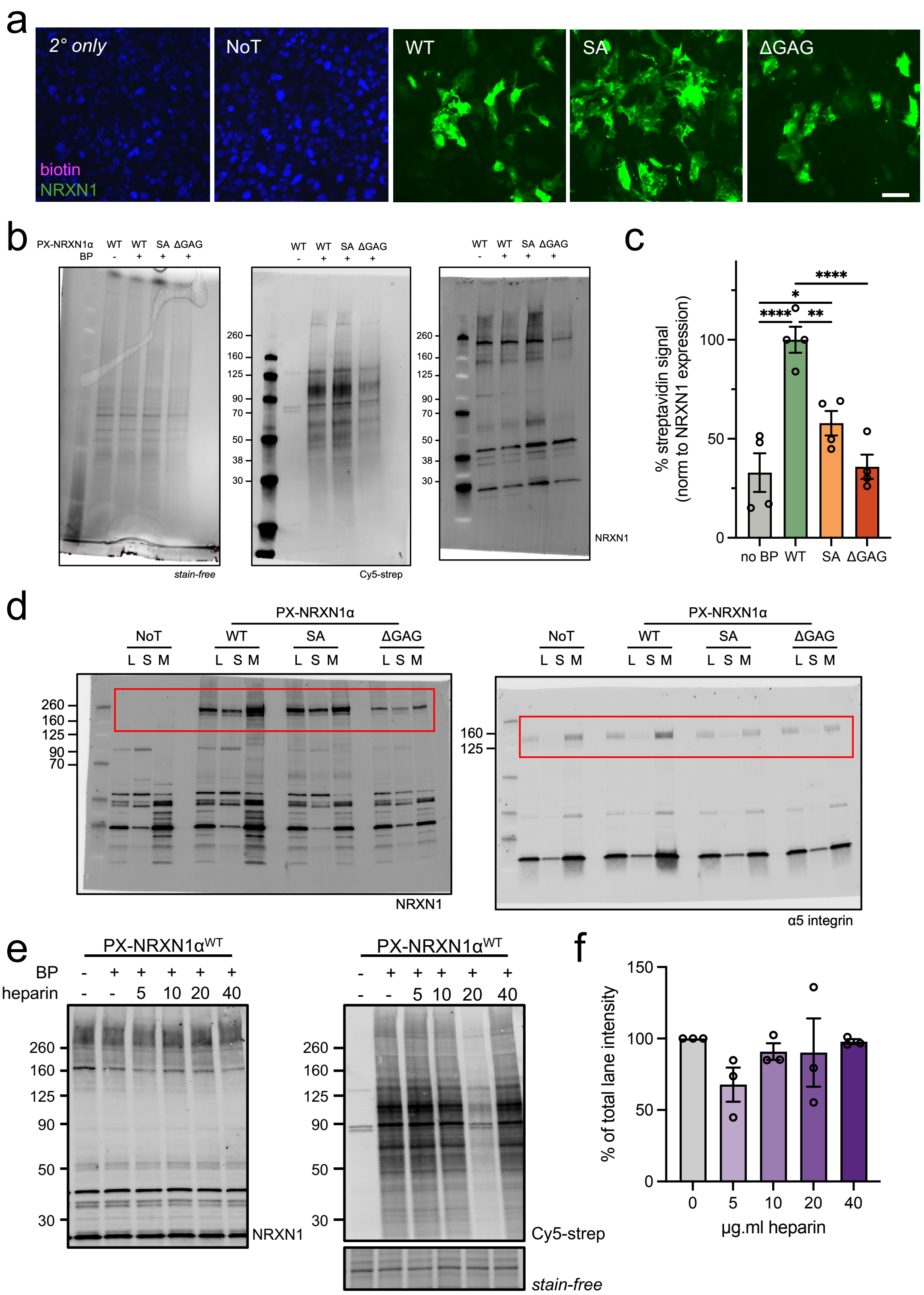

Supplemental Figure 2. Optimization of proximity labeling for identification of GAG-mediated interactions. (**a**) Additional microscopy panels showing non-transfected cells (NoT) and PX-NRNX1α^{WT} transfected cells without primary antibody (NRNX1) incubation. Scale bar = 75 μm. (**b**) Uncropped images of Figure 2b. (**d**) Uncropped images of Figure 2d, areas used highlighted in red. (**c**) Quantification biotinylation signal when normalized to NRXN1 signal in Fig 2b-c. (**d**) Uncropped images of Figure 2d. We note that non-specific bands are present in our western blot analysis, but these do not overlap with the expected of molecular weight range of PX-NRXN1α. (**e,f**) Pre-incubation of cells with heparin results in a limited decrease in biotinylation as observed by (**e**) western blot. (**f**) These

observations were highly variable between experiments, with only 5 and 20 μg/ml resulting in any decrease in biotinylation. Data representative of two biological replicates. Bar graphs represent means, and error bars represent SEM.

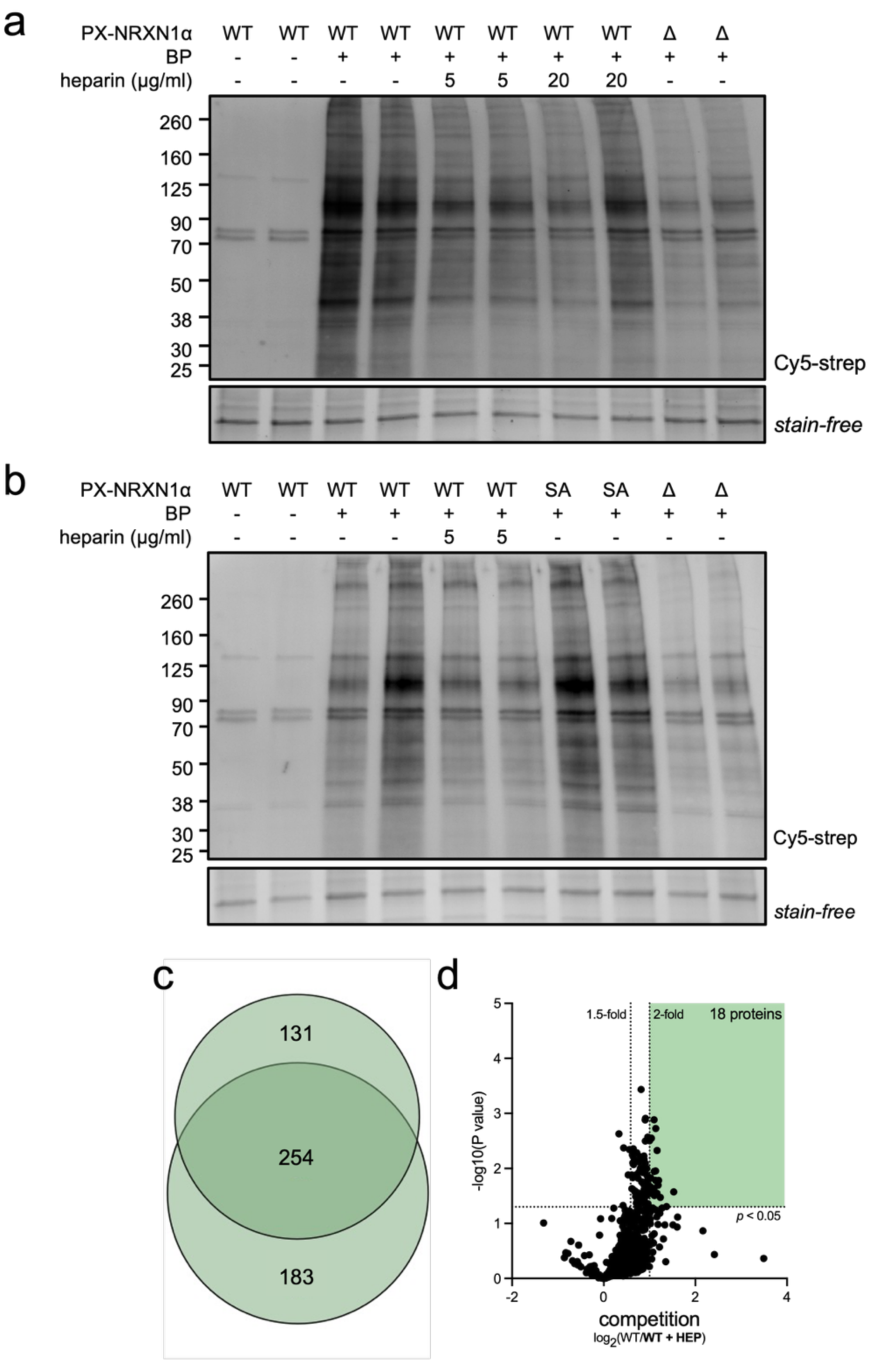

Supplemental Figure 3. Additional PX-NRNX1α proteomics analysis. (**a, b**) Western blot analysis of biological replicate samples prepared for proteomics analysis. (**c**) A total of 568 significantly enriched (TMT ratio of PX-NRNX1α^{WT} / PX-NRNX1α^{WT} – BP ≥ 5) proteins were identified across two biological replicates. (**d**) Volcano plot of proteins significantly enriched ($p < 0.05$) and 2-fold competed (TMT ratio of PX-NRNX1α^{WT} / PX-NRNX1α^{WT} + 5 μg/mL heparin ≥ 2).

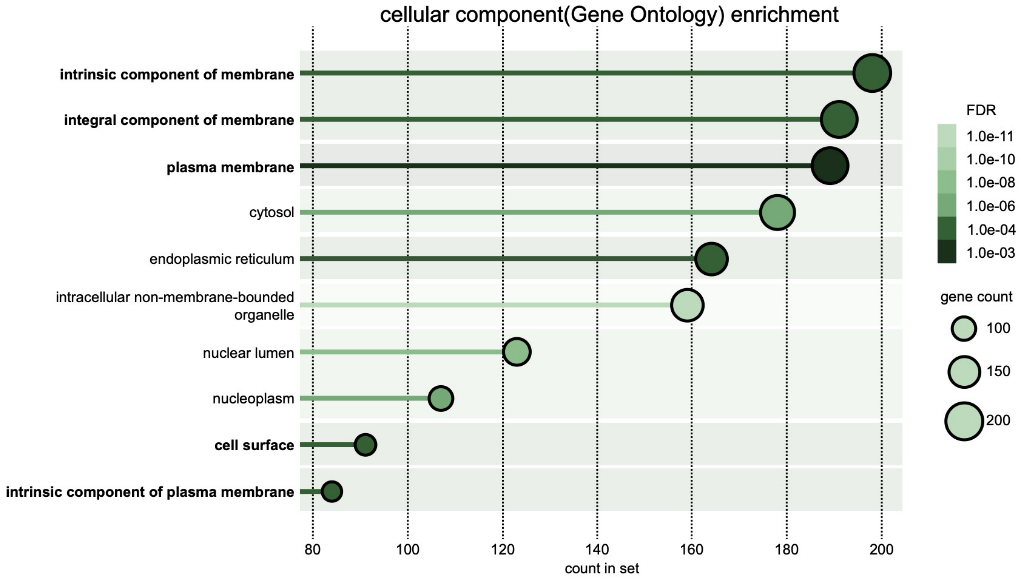

Supplemental Figure S4. Gene Ontology analysis of proteomic hits in **Figure 3**.

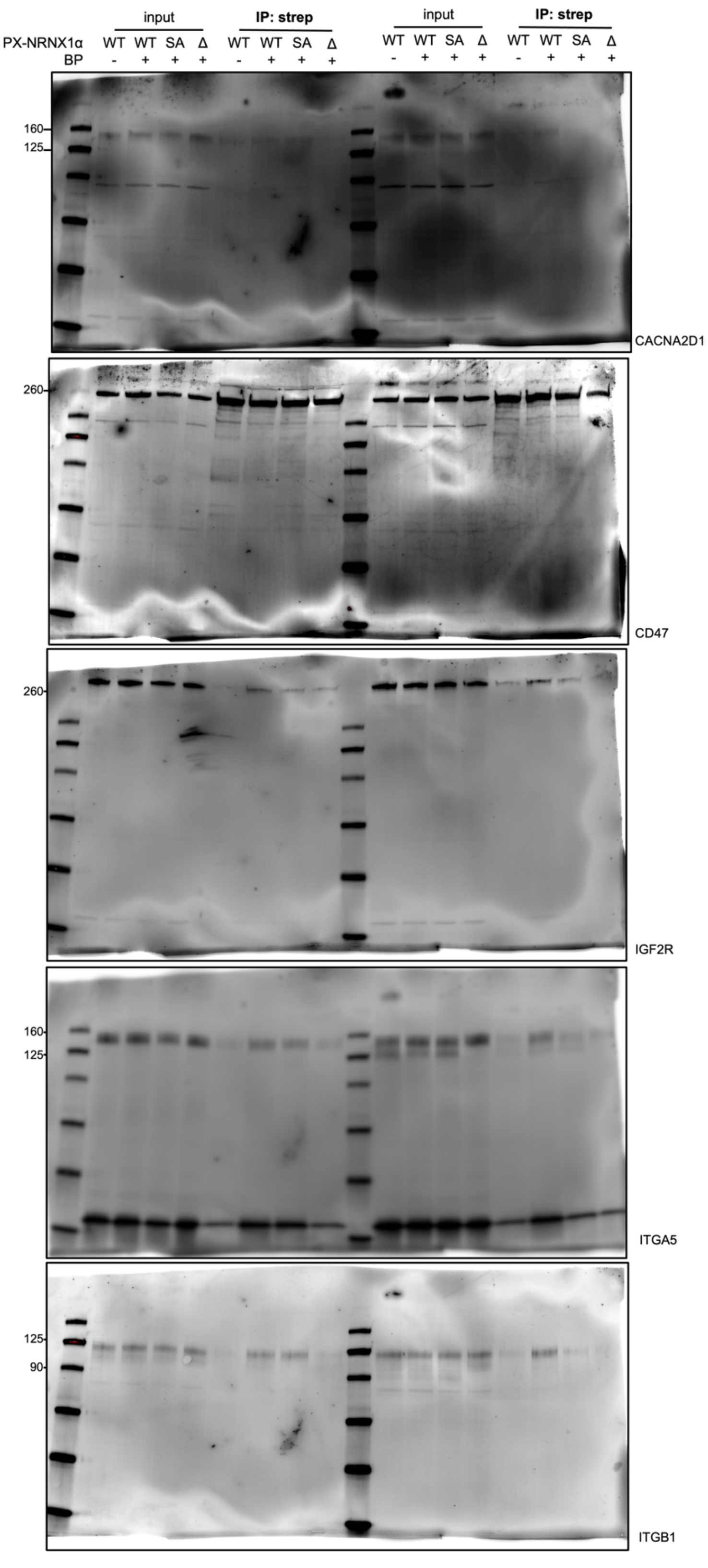

Supplemental Figure 5. Uncropped western blots for streptavidin enrichment. From top: CACNA2D1, CD47, IGF2R, ITGA5, ITGB1. Blot depicts two independent biological replicates.
